# Supplementary material for: Shrub Cover and Soil Moisture Affect Taxus baccata L. Regeneration at Its Southern Range
Source: Plants (Basel). 2023 Apr 28;12(9):1819. doi: 10.3390/plants12091819 (PMC10181193; doi:10.3390/plants12091819)
Supplement: Supplementary file 1 [file plants-12-01819-s001.zip › Figure S1 R Script.pdf]

```
### R script for analysis of taxus regeneration on 40 plots:
rm(list=ls())
require(AICcmodavg)
require(lmtest)
data<-read.table("C:\\Data\\Taxus40d.txt", header=T)
names(data)
summary(data)
attach(data)
Recr<-(Sap+Seed)
SR<-Fem/(Fem+Male)
Shrub<-(Shr+Spi)
range(Recr)
boxplot(Recr)

## univariate model selection of the variable above
M0<-glm(formula = Recr ~ 1, data = data, family = poisson)
M1<-glm(formula = Recr ~ aDBH, data = data, family = poisson)
M2<-glm(formula = Recr ~ Morph, data = data, family = poisson)
M3<-glm(formula = Recr ~ Pend, data = data, family = poisson)
M4<-glm(formula = Recr ~ Ssmo, data = data, family = poisson)
M5<-glm(formula = Recr ~ Bro, data = data, family = poisson)
M6<-glm(formula = Recr ~ Clos, data = data, family = poisson)
M7<-glm(formula = Recr ~ SR, data = data, family = poisson)
M8<-glm(formula = Recr ~ Shrub, data = data, family = poisson)

## model selection table based on AICc
fits <- list(M1,M2,M3,M4,M5,M6,M7,M8,M0)
names <- c("MaDBH","MMorph","MPend","MSsmo","MBro","MClos","MSR","MShrub","M0")
Table1<-aictab(cand.set = fits, modnames = names, sort = TRUE)
Table1

## estimating the deviance explained of best univariate models
(deviance(M0)-deviance(M8))/deviance(M0)
(deviance(M0)-deviance(M7))/deviance(M0)
(deviance(M0)-deviance(M1))/deviance(M0)
(deviance(M0)-deviance(M2))/deviance(M0)
(deviance(M0)-deviance(M5))/deviance(M0)
(deviance(M0)-deviance(M4))/deviance(M0)

## estimating c.hat from best model, 1.27, i.e. AICc is OK.
c_hat(M8, method = "deviance")
## model selection based on QAICc does not change.
Table1Q<-aictab(cand.set = fits, modnames = names, sort = TRUE, c.hat=1.27)
Table1Q

## M8, M7, M1, M2, M5, M4 are clearly better than M0.
## Considering results of Table 1, below the multivariate backward selection, taking out the least
significant variable at a time.
```

```
MFull<-glm(formula = Recr ~ Shrub + SR + aDBH + Morph + Bro + Ssmo, data = data, family =
poisson)
summary(MFull)
## is MFull an appropriated model? Estimate the five-percent critical value for a chi-squared
qchisq(0.95, df.residual(MFull))
pr <- residuals(MFull,"pearson")
sum(pr^2)
## Residual deviance (39.247) and sum of pearson (36.70) of MFull are lower that the critical value
(47.40)
## estimating c.hat of general model
c_hat(MFull, method = "deviance")
## result is c.hat = 1.19, i.e. AICc is OK.

## Multivariate model selection
M1b<-glm(formula = Recr ~ Shrub + aDBH + Morph + Bro + Ssmo, data = data, family = poisson)
M2b<-glm(formula = Recr ~ Shrub + aDBH + Morph + Bro, data = data, family = poisson)
M3b<-glm(formula = Recr ~ Shrub + Morph + Bro, data = data, family = poisson)
M4b<-glm(formula = Recr ~ Shrub + Morph, data = data, family = poisson)
M5b<-glm(formula = Recr ~ Shrub + Bro, data = data, family = poisson)
M6b<-glm(formula = Recr ~ Morph + Bro, data = data, family = poisson)
## model selection tabel based on AICc
fits <- list(MFull,M1b,M2b,M3b,M4b,M5b,M6b,M8,M2,M5,M0)
names <- c("MFull","M1b","M2b","M3b","M4b","M5b","M6b","M8","M2","M5","M0")
Table2<-aictab(cand.set = fits, modnames = names, sort = TRUE)
Table2
lrtest(M3b,M4b)
lrtest(M3b,M2b)
lrtest(M3b,M8)
## based on LRT we keep best model M3b
summary(M3b)
summary(M8)
summary(M2)
summary(M5)

## estimating the deviance explained of multivariate models
(deviance(M0)-deviance(M3b))/deviance(M0)
(deviance(M0)-deviance(M4b))/deviance(M0)
(deviance(M0)-deviance(M2b))/deviance(M0)
(deviance(M0)-deviance(M8))/deviance(M0)
(deviance(M0)-deviance(M1b))/deviance(M0)
(deviance(M0)-deviance(M5b))/deviance(M0)
(deviance(M0)-deviance(MFull))/deviance(M0)
(deviance(M0)-deviance(M6b))/deviance(M0)
(deviance(M0)-deviance(M2))/deviance(M0)
(deviance(M0)-deviance(M5))/deviance(M0)


## the sign of Bro inverts in multivariate (+, while in univariate is -)
```

```
## it could be determined by interactions with Shrub or Morph.
boxplot(Shrub~Bro, data=data, main="Shrub cover decrease with browsing", ylab="Shrub cover")
boxplot(Shrub~Morph, data=data, main="Shrub cover increases nearby watercourses",
ylab="Shrub cover")
M3bI<-glm(formula = Recr ~ Shrub + Morph + Bro + Bro*Shrub + Morph*Shrub, data = data, family
= poisson)
summary(M3bI)
## with the interaction the effect of Bro is negative again
lrtest(M3bI,M3b)
## there is no significant difference

## interactions can be assessed with automated model selection, using glmulti
## first, we perform automated model selection starting from the variable retained in MFull,
without interactions
require(glmulti)
output1<-glmulti("Recr",c("Shrub","SR","aDBH","Morph","Bro","Ssmo"),
data=data,level=1,intercept=TRUE,crit=aicc,family=poisson)
summary(output1)
weightable(output1)
coef(output1)
## best model is "Recr ~ 1 + Morph + Bro + Shrub", same result as from the backward model
selection above.
## to assess the effect of interactions on parameter estimates, we perform automated model
selection starting from the 3 variables retained in best model M3b
## interactions were allowed (level=2) with marginality true
output2<-glmulti("Recr",c("Shrub","Morph","Bro"),
data=data,level=2,marginality=TRUE,intercept=TRUE,crit=aicc,family=poisson)
summary(output2)
weightable(output2)
coef(output2)
## again, best model is Recr ~ 1 + Morph + Bro + Shrub
## however, several models with interactions have similar weight
## coef(output2) hows that the effect of Bro is negative, while Shrub and Morph (watercourse)
have positive effects

## is M3b an appropriated model? Estimate the five-percent critical value for a chi-squared
qchisq(0.95, df.residual(M3b))
pr <- residuals(M3b,"pearson")
sum(pr^2)
## Residual deviance (40.348) and sum of pearson (37.654) of M2b are lower that the critical value
(50,998)

## explanatory power
## R2 cannot be estimated with glm, instead we can calculate the analogous "deviance explained"
## estimating the deviance explained of the best model with no interactions
((deviance(M0)-deviance(M3b))/deviance(M0))
## deviance explained = 0.716, showing very good explanatory power
```

```
## plotting Recr vs Shrubs poisson
range(Shrub)
xShrub <- seq(1, 8, 1)
yRecr <- predict(M8, list(Shrub = xShrub),type="response")
plot(Shrub, Recr, pch = 20, xlab = "relative abundance of shrubs", ylab = "Common yew natural
regeneration", cex.lab=1.25)
lines(xShrub, yRecr)
```
