# Supplementary material for: Shrub Cover and Soil Moisture Affect Taxus baccata L. Regeneration at Its Southern Range
Source: Plants (Basel). 2023 Apr 28;12(9):1819. doi: 10.3390/plants12091819 (PMC10181193; doi:10.3390/plants12091819)
Supplement: Supplementary file 1 [file plants-12-01819-s001.zip › Table S1.pdf]

Table S1. List of the studied sites, their location, coordinates (Coord.), lithology (Bas = basalts; Gra = granitoids; Grd = granodiorites; Lim = Limestones; Met = metamorphic rocks), elevation (Elev.), and number of plots for searching recruitment. In the column Plots, the statement "all" means that all the site has been checked.

code	Zone	Locality	Municipality	Coord. X	Coord. Y	Lithology	Elev. (m a.s.l.)	Plots
**1**	**Gennargentu**	Rio Aratu (Cuile S'Arena)	Desulo	4429965.68	524514.86	Met	1000-1500	2
**2**	**Gennargentu**	Funtana S'Enis (Riu Correboi)	Fonni/Villagrande S.	4434953.89	529090.74	Grd	1220-1380	3
**3**	**Gennargentu**	Cuile Mattarano	Arzana	4423401.94	530028.84	Grd	1080-1100	all
**4**	**Supr. Ba. Ur.**	Su Clovu	Baunei	4440926.51	549738.75	Lim	800-815	all
**5**	**Gennargentu**	Bacu Searthu (Rio Is Enis)	Villagrande Strisaili	4428453.29	528739.61	Met	1200-1250	all
**6**	**Supr. Ba. Ur.**	Bacu Addas	Baunei	4443448.22	550511.05	Lim	575-815	2
**7**	**Marghine**	Mularza Noa	Bolotana	4467473.85	492382.21	Bas	925-975	4
**8**	**Gennargentu**	Bacu Sa Mandara	Villagrande Strisaili	4434900.87	534512.49	Met	1100-1300	all
**9**	**Gennargentu**	Fun. Ortu is Arangios	Desulo/Arzana	4426807.76	527057.69	Gra	1450-1650	4
**10**	**Supr. Ba. Ur.**	S'Eni Pidargiu	Baunei	4441941.45	549299.43	Lim	880-900	all
**11**	**Supr. Ba. Ur.**	Gorroppeddu	Baunei/Urzulei	4444073.28	548113.99	Lim	770-790	all
**12**	**Supr. Ol. Or. Ur**	M. Novo S. Giovanni	Orgosolo	4441061.81	535337.22	Lim	1150-1250	2
**13**	**Supr. Ol. Or. Ur**	Riu S'Eni	Orgosolo	4441083.20	537149.01	Lim	880-1000	all
**14**	**Ogliastra**	Baccu Is Enis	Villagrande Strisaili	4425022.46	540808.37	Met	900-1000	all
**15**	**Catena del Linas**	Canali 'e Nesta	Gonnosfanadiga	4366499.67	467928.70	Gra	875-925	all
**16**	**Supr. Ol. Or. Ur**	Monte Fumai	Orgosolo	4439778.13	534058.66	Lim	1135-1285	2
**17**	**Montalbo Lula**	Sa Marmurata	Lula	4480001.45	544856.07	Lim	730-770	all
**18**	**Limbara**	P. Berritta (Valle Suprappare)	Berchidda	4521812.33	514725.33	Gra	1100-1350	3
**19**	**Supr. Ol. Or. Ur**	Su Suercone	Orgosolo	4450898.69	540725.44	Bas	690-850	3
**20**	**Goceano**	Sos Nibberos	Bono	4474704.18	499667.91	Met	950-1100	5
**21**	**Montalbo Lula**	S'Ortu 'e Trullìo	Lula	4479573.54	544357.29	Lim	850-920	all
**22**	**Ogliastra**	Su Candelassargiu	Gairo	4413262.05	539209.76	Lim	950-1020	3
**23**	**Monti del Sulcis**	Canale Longufresu	Uta	4333134.82	486580.45	Gra	815-900	all
**24**	**M. di Alà**	Ferulas (Tassu Mannu)	Alà dei Sardi	4505089.50	524355.92	Gra	930-1010	all
**25**	**Barbagia Seulo**	Rio Longufresu (Stampu Turrunu)	Seulo/Sadali	4411121.58	522656.55	Lim	750-900	4
**26**	**Ogliastra**	Rio Taquisara	Gairo/Ussassai	4410059.66	538644.05	Lim	720-750	all
**27**	**Montalbo Lula**	Su Renagliu	Lula	4479336.81	544107.03	Lim	850-1010	all
**28**	**M. Nieddu Gal.**	S'Inferru	San Teodoro	4509821.26	548552.41	Gra	470-600	all
**29**	**Marghine**	Rio Biralotta	Bolotana	4467122.61	492323.45	Bas	1000-1020	3
**30**	**Lerno**	Rodè	Pattada	4495729.55	513584.56	Gra	850-900	all
**31**	**M. Nieddu Gal.**	Rio Badde Niedda	Padru	4512162.56	548449.86	Gra	270-500	2
**32**	**Monti del Sulcis**	Canale Longuvresu	Pula	4320046.36	490859.66	Gra	535-555	all
**33**	**Ogliastra**	Rio Ermolinus	Seui	4413476.21	533629.79	Lim	800-1100	5
**34**	**Goceano**	Funtana Tassu (Nuraghe Su Tassu)	Bultei	4484445.08	505586.34	Met	940-980	4
**35**	**Limbara**	Riu di Li Tassi	Tempio	4519283.38	504768.00	Gra	530-610	all
**36**	**Sarcidano**	Funtana Mela (Rio Bau Onu)	Laconi	4413936.19	508007.54	Lim	650-750	5
**37**	**Limbara**	Rio Rizzolu 'e Curadore	Oschiri/Tempio	4520055.63	507713.74	Met	430-630	5
**38**	**Montiferru**	Rio S'Abba Lughida	Cuglieri/S. Lussurgiu	4445650.43	467208.66	Bas	850-890	3
**39**	**Limbara**	P. Bandera NW (Funtana Bandera)	Tempio/Calangianus	4522636.70	515748.78	Gra	1200-1330	5
**40**	**Limbara**	Ghjroni (M. Niiddoni. NE)	Calangianus	4524101.23	517679.64	Gra	850-1000	5
