# Supplementary material for: Shrub Cover and Soil Moisture Affect Taxus baccata L. Regeneration at Its Southern Range
Source: Plants (Basel). 2023 Apr 28;12(9):1819. doi: 10.3390/plants12091819 (PMC10181193; doi:10.3390/plants12091819)
Supplement: Supplementary file 1 [file plants-12-01819-s001.zip › Table S2.pdf]

Table S2. Variables used in this study and their values. nAT= number of adult trees; Dmax= maximum DBH; Daver= average DBH; Fem= female yews; Male= male yews; Sap= saplings; Seed = seedlings; Pend= declivity; Area=area of yew stands; Ssmo=soil summer moisture; Clos= closure of the canopy; Lay= layers of the canopy; Shr= shrubs; Spi= spiny shrubs; BroL= browsing by livestock; BroW= browsing by wild animals; BroT= total browsing.

code	Area	nAT	Dmax	Daver	Fem	Male	Sap	Seed	Pend	Ssmo	Lay	Clos	Shr	Spi	ShrT	BroL	BroW	BroT
1	2.5	58	1840	1304	11	16	0	0	30	Yes	2	60	0	1	1	Yes	Yes	Yes
2	1	32	1750	905	15	16	3	2	21	Yes	2	50	3	2	3	Yes	Yes	Yes
3	1.2	30	2000	900	12	18	0	0	12.5	Yes	2	75	0	1	1	Yes	Yes	Yes
4	0.6	16	1000	745	6	10	1	0	22.5	No	1	50	1	0	1	Yes	Yes	Yes
5	0.5	23	1750	700	5	8	0	0	15	Yes	2	60	1	1	1	Yes	Yes	Yes
6	0.5	23	1050	682	8	15	0	0	24	No	1	50	2	0	1	Yes	Yes	Yes
7	3.2	181	1650	658	47	55	0	0	20	Yes	2	80	0	2	1	No	No	No
8	1	21	1500	650	6	13	0	0	15	Yes	1	75	0	0	0	Yes	Yes	Yes
9	7.5	182	1080	636	69	63	0	0	20	Yes	1	55	0	0	0	Yes	Yes	Yes
10	0.8	15	1500	557	5	10	2	2	7.5	No	2	50	3	2	3	Yes	Yes	Yes
11	0.6	21	1210	534	9	12	3	2	5	No	1	80	1	3	2	Yes	Yes	Yes
12	1.2	78	800	523	17	12	2	3	30	No	1	65	2	2	2	Yes	Yes	Yes
13	3	24	1300	520	9	15	2	0	20	No	2	70	0	2	1	Yes	Yes	Yes
14	0.5	36	1200	500	8	15	2	0	20	Yes	3	80	1	2	2	No	No	No
15	0.4	17	1200	490	3	14	0	0	30	Yes	1	80	0	0	0	No	Yes	Yes
16	1	31	790	488	13	11	2	0	42.5	No	1	60	3	1	2	Yes	Yes	Yes
17	0.5	42	820	425	21	19	0	0	35	No	1	70	0	1	1	Yes	Yes	Yes
18	3.5	52	1220	404	26	21	3	2	42.5	No	2	60	5	3	4	No	No	No
19	4.3	158	1224	384	45	69	0	0	45	No	1	60	0	1	1	No	Yes	Yes
20	6.6	836	1690	352	263	573	0	0	11	Yes	1	90	0	2	1	Yes	No	Yes
21	1.5	48	840	350	18	29	1	0	35	No	1	90	0	1	1	Yes	Yes	Yes
22	1.2	58	570	345	19	30	0	0	10	No	1	75	0	0	0	Yes	Yes	Yes
23	0.8	23	780	321	9	14	1	0	40	Yes	1	90	1	1	1	No	Yes	Yes
24	0.5	25	790	317	12	13	2	0	25	Yes	2	85	3	3	3	Yes	Yes	Yes
25	3	93	1110	314	30	43	3	0	12.5	Yes	2	80	2	2	2	Yes	No	Yes
26	1	19	640	302	8	8	2	2	3.5	Yes	2	70	3	3	3	No	No	No
27	4	57	650	300	23	27	2	0	30	No	1	90	2	2	2	Yes	Yes	Yes
28	3	77	1005	289	27	32	4	2	40	Yes	3	70	4	3	4	No	No	No
29	7.8	45	1120	281	19	21	0	0	2.5	No	2	85	0	1	1	Yes	No	Yes
30	0.4	17	570	267	7	10	0	0	30	Yes	2	90	1	2	2	No	Yes	Yes
31	2.5	54	500	236	12	11	5	3	30	Yes	3	85	2	4	3	No	No	No
32	0.5	11	460	224	1	3	0	0	45	No	1	70	0	0	0	No	Yes	Yes
33	10	198	650	215	58	71	4	3	23.5	Yes	2	80	3	2	3	No	Yes	Yes
34	4.5	103	400	210	42	43	3	3	5	Yes	1	75	2	3	3	Yes	Yes	Yes
35	1.5	35	900	183	18	11	3	3	22.5	Yes	2	85	2	4	3	No	No	No
36	5	115	560	182	34	29	4	2	20	Yes	3	80	3	3	3	No	No	No
37	7.8	135	950	180	62	35	4	2	27.5	Yes	3	80	3	5	4	No	No	No
38	2	26	380	151	7	6	3	0	20	Yes	2	85	2	3	3	No	Yes	Yes
39	3	368	930	145	143	166	5	4	34	Yes	2	55	4	4	4	No	No	No
40	4.5	293	660	138	126	114	5	4	22.5	Yes	2	85	2	4	3	No	No	No
